# Knowledge of cancer symptoms and risk factors: A cross-sectional study from a developing country

**DOI:** 10.1097/MD.0000000000037823

**Published:** 2024-04-12

**Authors:** Samir Al Bashir, Majd M. AlBarakat, Karam K. Alabedalhalim, Ali Al-Khalaileh, Amer Alassaf, Othman Saleh, Asmaa W. Ayyad, Karem H. Alzoubi

**Affiliations:** a Department of Pathology and Microbiology, Faculty of Medicine, Jordan University of Science and Technology, Irbid, Jordan; b Faculty of Medicine, Jordan University of Science and Technology, Irbid, Jordan; c Faculty of Medicine, The Hashemite University, Zarqa, Jordan; d Branch of Otolaryngology - Department of Special Surgery, Faculty of Medicine, Jordan University of Science and Technology, Irbid, Jordan; e Department of Pharmacy Practice and Pharmacotherapeutics, College of Pharmacy, University of Sharjah, Sharjah, UAE; f Department of Clinical Pharmacy, Faculty of Pharmacy, Jordan University of Science and Technology, Irbid, Jordan.

**Keywords:** cancer awareness, cancer risk factors, cancer signs and symptoms, early presentation, low- and middle-income countries

## Abstract

The delayed presentation of cancer patients to healthcare facilities for diagnosis is a pressing issue, as late-stage cancer cases are often more challenging to treat effectively. In low-resource settings, such as those with limited access to healthcare facilities, the impact of inadequate awareness of cancer signs and symptoms can be particularly severe. Therefore, this study aimed to evaluate public knowledge of cancer signs and symptoms and risk factors in the context of Jordan. This cross-sectional study was conducted among participants from all settings. Data was obtained from an Arabic version of the cancer awareness measure (CAM), which was answered online. It described demographic data and knowledge of cancer prevalence, age-related risk, signs, symptoms, and risk factors in recall and recognition-type questions. Participants (n = 1998) completed the questionnaire with a Median age of 35 and an interquartile range of 24. About half (n = 1070) thought that cancer is unrelated to age. Most participants identified breast cancer as the most common cancer among women (81%). Awareness of cancer signs/symptoms significantly differed in the level of knowledge in favor of females. The symptom “unexplained weight loss” was most commonly recognized (66.3%) and “persistent difficulty swallowing” the least (42.6%). As for risk factors, “smoking” was the most identified (76.9%) and “eating less than 5 portions of fruits and vegetables a day” was the least (19%), and “doing <30 minutes of moderate physical activity 5 times a week” as a close second least (19.95%). Females identified “smoking,” “passive smoking,” “HPV infection,” and “having a close relative with cancer” as risk factors significantly more than males. Those with good economic status were more aware that smoking is a cancer risk factor by 1.51 times compared to those with poor economic status. To enhance early diagnosis and presentation in Jordan, there is a need for increased public awareness of the signs, symptoms, and risk factors of cancer. One effective strategy to achieve this goal is to conduct targeted public campaigns that cater to different population groups, such as the youth, to improve their understanding and ensure that the message resonates.

## 1. Introduction

Cancer is a significant health concern in Jordan, with increasing incidence rates in recent years. According to the Jordan Cancer Registry 2020 report, there were 11,559 newly diagnosed cancer cases among both sexes and all ages. This number has been steadily increasing over the years, with a 3.3% annual percentage change observed between 2006 and 2016.^[1]^ The most commonly diagnosed cancers in Jordan are breast, colorectal, lymphoma, lung, and thyroid cancers.^[1]^

Lung cancer has the highest mortality rate in Jordan, accounting for 15.2% of cancer-related deaths due to the high prevalence of smoking in the country.^[1]^ Overall, cancer is the second leading cause of death in Jordan, following ischemic heart disease.^[2]^ The cancer mortality rate in Jordan is high at 15%, highlighting the urgent need for effective prevention and early detection strategies.^[3]^

Several factors contribute to the high incidence of cancer in Jordan, including changes in lifestyle and an aging population. Smoking, poor diet, and physical inactivity are major risk factors for cancer in Jordan.^[4]^ Additionally, environmental factors such as pollution and exposure to carcinogens in the workplace can increase cancer risk.

To address the rising incidence of cancer in Jordan, several initiatives have been implemented. These include raising public awareness about cancer risk factors and symptoms, improving cancer screening and early detection programs, and promoting healthy lifestyles.^[5]^ The Jordanian Ministry of Health has also established cancer centers throughout the country to provide comprehensive cancer care to patients.

In conclusion, cancer incidence is increasing in Jordan, with lung, breast, colorectal, lymphoma, and thyroid cancers being the most commonly diagnosed. The high prevalence of risk factors such as smoking, poor diet, and physical inactivity highlights the importance of promoting healthy lifestyles and effective prevention strategies. With continued efforts to improve cancer care and early detection in Jordan, it is hoped that the burden of cancer can be reduced in the future.

## 2. Method

### 2.1. Study design and settings

This cross-sectional observational survey was carried out in Jordan between January and May 2023 to assess Jordanians’ knowledge about cancer symptoms and its risk factors. People who were eligible to participate in the study were adult Jordanians. The study survey was uploaded to Google Forms and distributed via social media platforms. The protocol of this study was approved by the Institutional Review Board of Jordan University of Science and Technology (Approval # 32/159/2023). Electronic informed consent was obtained from all study participants.

On the first page of the online questionnaire, participants were informed about the study aim, objective, and right to withdraw at any point. Also, they were assured that all information would be confidential and used for research purposes only.

### 2.2. Sample size determination

To determine the estimated sample size to be included in this online survey, the online sample size calculator Raosoft was used with an accepted margin of error of 5%, a confidence interval of 95%, and a response distribution of 50%. The minimal sample size calculated was 385 participants of the total population, which was 11150000 people.

### 2.3. Steady measures

The cancer awareness measure (CAM) questionnaire, a validated tool for measuring cancer awareness in the public, was employed as an assessment tool.^[6]^ The CAM was translated from English to Arabic by 2 healthcare professionals who were proficient in both languages and had experience in health survey design and was subsequently used in our study. It comprises 4 sections, the first of which details the demographic characteristics of the participants. The second section evaluated Jordanians’ awareness of cancer prevalence and age-specific risk. The third section included open-ended (recall) questions, while the fourth section contained closed (recognition) questions, enabling a comparison between these 2 types of questions (recall vs recognition).

### 2.4. Data collection

To recruit participants, an online Arabic form was disseminated through social media platforms such as Facebook, Telegram, and WhatsApp. The survey began with a standard question that asked participants if they agreed to participate in the study. Upon answering yes, participants could proceed to the comprehensive study questions that took approximately 10 to 15 minutes to complete. The questionnaire gathered information on personal behavioral characteristics such as age, gender, education level, and income. To assess cancer symptom awareness, a list of 8 symptoms was presented, each categorized as a cancer warning sign or not.

### 2.5. Pilot study

Pilot research on 15 respondents was undertaken before data collection to examine the validity and reliability of the Arabic version of the CAM Questionnaire. The pilot study was also used to ensure that the questionnaire was clear. The pilot standardized Cronbach Alpha of the Arabic Version of the CAM questionnaire was > 0.65.

### 2.6. Statistical analysis

All data were entered into an Excel spreadsheet and then exported to the statistical program SPSS version (24, IBM SPSS, Chicago, IL). Data were cleaned using frequency and tabulation to verify accuracy, consistency, and missing values. The significance threshold was set at a *P* value of <.05 for all statistical tests employed in this study. Descriptive statistics were used for sociodemographic data, and univariate analysis was used to analyze factors influencing participants’ knowledge level using the Mann-Whitney U-test (for non-normal continuous variables), t-test (for normal distribution of continuous data), and chi-squared test (for categorical variables). Then, a multivariate logistic regression analysis was conducted for the variables with significance (*P* < .05) in the univariate analysis to evaluate the odds ratios of the factors.

## 3. Results

The survey was fully completed by 1998 individuals, with a median age in years of 35.0 and an interquartile range of 24.0. Approximately half of the participants were female (53.1%), and the majority resided in urban areas (82.2%). Of the respondents, 54.6% were unmarried, and 64.1% had earned a bachelor, master, or Ph.D. degree (Table 1).

**Table 1:** Socioeconomic characteristics of the participants.

Variables		N (%)
Age	Median (IQR)	35.0 (24.0)*
Gender	Male	935 (46.9%)
	Female	1059 (53.1%)
Living place	Urban	1638 (82.2)
	Rural area	354 (17.8)
Social status	Singe	1055 (54.6)
	Married	444 (22.9)
	Widowed	374 (19.4)
	Divorced	119 (6.2)
Educational level	No education	12 (0.6)
	Primary school	90 (4.6%)
	High school	444 (22.9%)
	College	150 (7.7%)
	bachelor's or master degree or PhD degree	1242 (64.1%)
Working status	Student or not working	1398 (70.6)
	Job	420 (21.3)
	Own a business or work from home	108 (5.4)
	Retired	54 (2.7)
Economic status	Poor	600 (30.3)
	Good	1380 (69.7)
Did you have cancer?	Yes	84 (4.9)
	No	1608 (94)
	I do not know	18 (1.1)
Did one of your wife/husband have cancer?	Yes	30 (1.8)
	No	1578 (97)
	I do not know	18 (1.1)
Did one of your family have cancer?	Yes	690 (37)
	No	1140 (61.1)
	I do not know	36 (1.9)
Did one of your friends have cancer?	Yes	336 (19.9)
	No	1308 (77.3)
	I do not know	48

* The data represented by median (IQR).

Of the participants, 1070 (53.5%) thought there was no relation between age and cancer (Table 2). However, females showed better recognition of the relationship between age and cancer (*P* < .001). Most participants (81.9%) said that breast cancer is the most common cancer among women, whilst nearly half of them (49.5%) reported that prostate cancer was the most common cancer among men. Both males and females showed similar knowledge of this aspect.

**Table 2:** Knowledge of cancer age-related risk and its prevalence.

Questions	Gender			Economic status			Living place		
	Male (N = 935)	Female (N = 1059)	*P* value	Poor (N = 600)	Good (N = 1380)	*P* value	Urban (N = 1638)	Rural (N = 354)	*P* value
Cancer is unrelated to age	465 (50.2%)	605 (58.3%)	<.001	378 (64.3%)	690 (50.7%)	<.001	4912 (56.7%)	162 (45.8%)	<.001
What do you think is the most common cancer in women?			<.001			<.001			<.001
Breast cancer	731 (78.8%)	907 (86.7%)		504 (84.8%)	1128 (82.5%)		1416 (87.7%)	222 (62.7%)	
Uterus cancer	90 (8.6%)	84 (9.1%)		60 (10.1%)	108 (7.9%)		96 (5.9%)	72 (20.3%)	
Lung cancer	50 (5.4%)	22 (2.1%)		18 (3.0%)	54 (3.9%)		48 (3.0%)	24 (6.8%)	
skin cancer	38 (4.1%)	4 (0.4%)		0 (0.0%)	42 (3.1%)		18 (1.1%)	24 (6.8%)	
Colon cancer	25 (2.7%)	23 (2.2%)		12 (2.0%)	36 (2.6%)		36 (2.2%)	12 (3.4%)	
What do you think is the most common cancer in men?			<.001			<.001			<.001
Prostate cancer	432 (47.2%)	558 (53.6%)		708 (52.2%)	276 (46.9%)		822 (50.9%)	162 (47.4%)	
Lung cancer	351 (38.3%)	369 (35.4%)		474 (35.0%)	240 (40.8%)		594 (36.8%)	126 (36.8%)	
Colon cancer	64 (7.0%)	58 (5.6%)		96 (7.1%)	24 (4.1%)		96 (5.9%)	30 (8.8%)	
Gastric cancer	44 (4.8%)	16 (1.5%)		30 (2.2%)	30 (5.1%)		36 (2.2%)	24 (7.0%)	
Urothelial Cancer	25 (2.7%)	41 (3.9%)		48 (3.5%)	18 (3.1%)		66 (4.1%)	0 (0.0%)	

Pearson Chi-squared test.

Unexplained weight loss was the most commonly recognized cancer symptom of cancer, with 66.3% of respondents identifying it (Table 3). This was followed by unexplained swelling/lump (63.6%) and unexplained pain (55.6%). The least recognized symptom was persistent difficulty swallowing, with only 42.6% of respondents identifying it as a potential sign of cancer.

**Table 3:** Awareness scores for cancer symptoms and signs in females versus males, people with poor economic status versus those with good economic status, and urban versus rural areas.

Signs/Symptoms	Gender			Economic status			Living place		
	Male (N = 935)	Female (N = 1059)	*P* value	Poor (N = 600)	Good (N = 1380)	*P* value	Urban (N = 1638)	Rural (N = 354)	*P* value
Unexplained swelling/lump	368 (39.4%)	891 (85.5%)	<.001	444 (75.5%)	1206 (88.2%)	<.001	1410 (87.4%)	246 (84.1%)	<.001
Unexplained pain	494 (53.5%)	594 (57.2%)	<.001	282 (48.7%)	804 (58.8%)	<.001	948 (59.0%)	144 (41.0%)	<.001
Unexplained bleeding	417 (45.9%)	570 (57.2%)	<.001	253 (45.8%)	726 (54.3%)	<.001	842 (53.9%)	143 (41.8%)	<.001
Persistent cough/hoarseness	406 (44.3%)	439 (44.6)	<.001	197 (35.9%)	642 (48.0%)	<.001	54 (15.5%)	263 (17.0%)	<.001
Persistent change in bowel habit	491 (53.1%)	580 (57.3%)	<.001	277 (48.9%)	792 (58.4%)	<.001	899 (56.7%)	176 (50.6%)	<.001
Persistent difficulty swallowing	371 (40.6%)	442 (44.6%)	<.001	410 (51.3%)	381 (57.4%)	<.001	497 (53.8%)	294 (54.5%)	<.001
Non-healing ulcer	419 (45.6%)	522 (52.3%)	.003	203 (36.3%)	738 (54.9%)	<.001	818 (52.2%)	123 (35.3%)	<.001
Unexplained weight loss	580 (62.9%)	713 (69.2%)	<.001	355 (62.2%)	924 (67.5%)	<.001	701 (75.9%)	406 (75.3%)	<.001
Change of appearance of a mole	373 (40.2%)	690 (68.4%)	<.001	984 (72.9%)	295 (52.3%)	<.001	1067 (67.6%)	218 (62.6%)	<.001

Pearson Chi-squared test.

Regarding gender differences, males were less likely than females to recognize unexplained swelling/lump, change in the appearance of a mole, and unexplained bleeding as cancer symptoms by 0.109, 0.286, and 0.639 times, respectively (Table 4). People with good economic status were more knowledgeable about all cancer symptoms (*P* < .001) except for “a change in the appearance of a mole.” In contrast, individuals with poor economic status were more aware (*P* < .001). Furthermore, those living in an urban were more likely to identify unexplained bleeding as a potential cancer symptom, with a 1.41 times higher likelihood than those living in rural areas.

**Table 4:** The association of gender, economic status, and place of living with knowledge about cancer signs/symptoms.

Signs/Symptoms	Male gender		Good economic status		Living in urban area	
	OR (95% CI)	*P* value	OR (95% CI)	*P* value	OR (95% CI)	*P* value
Unexplained swelling/lump	0.109 (0.0874–0.136)	<.001	1.607 (1.2710–2.031)	<.001	1.737 (1.3284–2.271)	<.001
Unexplained pain	1.127 (0.938–1.353)	.202	0.711 (0.583–0.868)	<.001	0.519 (0.408–0.659)	<.001
Unexplained bleeding	0.639 (0.532–0.769)	<.001	1.374 (1.121–1.684)	.002	1.453 (1.140–1.852)	.003
Persistent cough/hoarseness	0.981 (0.816–1.18)	.836	1.636 (1.330–2.01)	<.001	1.071 (0.841–1.36)	.576
Persistent change in bowel habit	0.832 (0.693–0.999)	.049	1.452 (1.189–1.772)	<.001	1.172 (0.924–1.487)	.190
Persistent difficulty swallowing	0.860 (0.715–1.04)	.111	1.412 (1.149–1.74)	.001	1.326 (1.033–1.70)	.027
Change of appearance of a mole	0.286 (0.236–0.348)	<.001	2.197 (1.777–2.716)	<.001	0.833 (0.648–1.069)	.151
Non-healing ulcer	0.772 (0.641–0.929)	.006	2.055 (1.671–2.526)	<.001	1.762 (1.376–2.257)	<.001
Unexplained weight loss	0.818 (0.674–0.992)	.041	1.134 (0.918–1.400)	.243	2.540 (1.999–3.228)	<.001

Overall, females exhibited a higher level of knowledge about cancer symptoms than males (Table 5). The most commonly recognized risks of cancer were smoking (93.5%), drinking alcohol (83.6%), and passive smoking (76.9%), whilst the least recognized was eating less than 5 portions of fruit and vegetables a day (19%. The second least was doing <30 minutes of moderate physical activity 5 times a week (19.95%). Males showed lower awareness than females in identifying that having a close relative with cancer (OR = 0.69, *P* < .001), hepatitis B virus infection (OR = 0.77, *P* = .009), smoking (0R = 0.30, *P* < .001) and passive smoking (OR = 0.74, *P* = .008) as risks factors of cancer. People with good economic status were more aware that smoking is a cancer risk factor by 1.51 times compared to people with a low economic status (Table 6).

**Table 5:** Awareness scores for cancer risk factors according to gender, economic status, and place of living.

Risk factors	Male gender			Good economic status			Living in urban area		
	Male (N = 935)	Female (N = 1059)	*P* value	Poor (N = 600)	Good (N = 1380)	*P* value	Urban (N = 1638)	Rural (N = 354)	*P* value
Smoking	823 (89.8%)	979 (96.7%)	<.001	522 (91.6%)	1266 (94.2%)	.035	1488 (94.3%)	312 (89.7%)	.002
Passive smoking	666 (72.9%)	810 (80.8%)	<.001	1026 (76.7%)	438 (77.7%)	.644	1284 (81.7%)	186 (54.4%)	<.001
Eating <5 portions of fruit and vegetables a day	181 (20.1%)	170 (18.5%)	.272^1^	132 (24.2%)	222 (16.9%)	<.001	300 (19.3%)	60 (17.9%)	.540
Being overweight (BMI > 25)	399 (44.1%)	417 (42.0%)	.337	228 (41.3%)	582 (43.7%)	.340	660 (42.5%)	156 (45.6%)	.288
Getting sunburnt more than once as a child	443 (48.9%)	469 (47.0%)	.407	155 (19.4%)	145 (21.8%)	.250^1^	768 (49.0%)	144 (42.9%)	.039
Being over 70 yr old	430 (47.7%)	440 (43.9%)	.100	384 (69.6%)	924 (70.3%)	<.745	720 (46.2%)	150 (43.9%)	.441
Having a close relative with cancer	590 (65.8%)	732 (74.4%)	<.001^1^	532 (66.6%)	484 (72.9%)	.009^1^	1116 (72.4%)	204 (60.7)	<.001
Doing <30 min of moderate physical activity 5 times a week	257 (28.4%)	255 (25.4%)	.140	353 (26.3%)	154 (27.8%)	.510	454 (28.9%)	58 (17.3)	<.001
HPV infection	443 (49.2%)	571 (57.6%)	<.001	756 (57.0%)	252 (45.7%)	<.001	912 (58.9%)	102 (29.8%)	<.001

Pearson Chi-squared test.

BMI = body-mass index, HPV = hepatitis B virus.

**Table 6:** The association of gender, economic status, and living site with knowledge about cancer risk factors.

Risk factors	Male gender		Good economic status		Living in urban area	
	OR (95% CI)	*P* value	OR (95% CI)	*P* value	OR (95% CI)	*P* value
Smoking	0.3036 (0.2011–0.458)	<.001	1.5171 (1.0335–2.227)	.033	1.5221 (1.0032–2.309)	.048
Passive smoking	0.740 (0.592–0.925)	.008	0.801 (0.625–1.027)	.081	3.776 (2.925–4.875)	<.001
Drinking more than 1 unit of alcohol a day	0.350 (0.269–0.456)	<.001	0.930 (0.703–1.230)	.609	2.114 (1.587–2.816)	<.001
Eating <5 portions of fruit and vegetables a day	1.196 (0.946–1.512)	.136	0.610 (0.477–0.781)	<.001	1.205 (0.881–1.649)	.244
Being overweight	1.081 (0.899–1.30)	.409	1.116 (0.911–1.37)	.290	0.874 (0.688–1.11)	.272
Getting sunburnt more than once as a child	1.077 (0.895–1.295)	.435	2.024 (1.648–2.486)	<.001	1.184 (0.927–1.513)	.176
Being over 70 yr old	1.150 (0.955–1.38)	.140	1.928 (1.567–2.37)	<.001	1.016 (0.797–1.30)	.895
Having a close relative with cancer	0.695 (0.568–0.851)	<.001	1.004 (0.806–1.252)	.969	1.581 (1.231–2.032)	<.001
HPV infection	0.776 (0.643–0.938)	.009	1.442 (1.173–1.774)	<.001	3.103 (2.401–4.011)	<.001
Doing <30 min of moderate physical activity 5 times a week	1.252 (1.018–1.54)	.033	0.859 (0.685–1.08)	.187	2.048 (1.505–2.79)	<.001

HPV = hepatitis B virus.

Table 7 shows that fear (64.9%) is the most common cause that some people choose not to go to the doctor even if serious cancer symptoms appear, whilst the least reported reason was people not wanting to waste the doctor time (15.1%). However, females were more likely to choose fear as a reason in comparison to males (*P* < .001). Moreover, difficulty in finding transport to go to the doctor was highly reported by people in rural areas compared to those who are city citizens (*P* < .001). Differences were found comparing the poor with the good economic status, such as difficulty in getting a meeting with the doctor, whereas people with poor economic status chose it more (*P* < .001).

**Table 7:** The reasons why some people do not go to the doctor even if serious symptoms appear.

The reasons	Male gender			Good economic status			Living in urban area		
	Male (N = 935)	Female (N = 1059)	*P* value	Poor (N = 600)	Good (N = 1380)	*P* value	Urban (N = 1638)	Rural (N = 354)	*P* value
The Embarrassment	186 (20.8%)	255 (25.9)	.002	141 (25.6%)	294 (22.4%)	.037	354 (23.0%)	81 (24.0%)	.166
The fear	505 (55.4%)	743 (73.7%)	<.001	366 (63.9%)	870 (65.3%)	.516	1041 (66.4%)	201 (57.6%)	<.001
I do not want to waste the doctor time	149 (16.6%)	135 (13.8%)	.179	168 (12.8)	116 (21.1%)	<.001	56 (16.6%)	228 (14.9%)	.004
It is difficult to talk with my doctor	254 (28.6%)	235 (23.9%)	<.001	159 (28.6)	330 (25.3%)	<.001	412 (26.9%)	77 (22.8%)	<.001
It is difficult to get a meeting with a doctor	302 (34.1%)	325 (33.3%)	.049	231 (41.3%)	390 (30.2%)	<.001	493 (32.4%)	128 (37.5%)	.025
I am very busy	395 (44.0)	467 (47.6)	.264	238 (42.8%)	612 (46.8%)	.004	717 (46.8%)	139 (40.5%)	.072
It is difficult to find a transport way to go to the doctor	449 (48.0%)	349 (33.1%)	<.001	562 (40.8%)	233 (39.0%)	.434	570 (34.9%)	228 (64.4%)	<.001

Pearson Chi-squared test.

## 4. Discussion

Having a fundamental comprehension of cancer risk factors and symptoms is essential for prompt recognition and seeking medical attention, particularly in low-resource settings such as rural areas where regular screening programs are not available, and early presentation serves as the primary method for early diagnosis. Inadequate cancer awareness can lead to delayed diagnosis and unfavorable outcomes. The main objective of this investigation was to evaluate Jordanians’ comprehension of cancer symptoms and risk factors. The overall outcomes revealed that age was not evenly dispersed among the study population. Unexplained weight loss was the most widely recognized symptom, while smoking was identified as the most significant risk factor. Fear was the most prevalent excuse for not consulting with a physician.

### 4.1. Age and related risk

The majority of participants believed that there was no relation between age and cancer. This might be because the sample was gathered through social media. Kyle et al findings were consistent with our results. They found that 66.9% of British teens thought cancer had nothing to do with age.^[7]^ Furthermore, in Gaza, a recent survey indicated that 65.3% of participants believed cancer was unrelated to age, and 3.9% were unsure if there was any relationship at all.^[8]^ This may imply a need for educational efforts on the relationship between age and cancer, as well as paying attention to early recognition of symptoms, particularly if they are felt at an advanced age because dismissing symptoms and discovering them at an older age is associated with an increased risk of cancer appearance and progression.^[9]^

#### 4.1.1. Recognized risk factors

Smoking, drinking, and passive smoking were the most generally known risk factors of cancer, whereas eating fewer than 5 portions of vegetables and fruits per day and completing <30 minutes of moderate physical activity 5 times per week were the least recognized. These findings are consistent with previous studies.^[10–13]^ This disparity may be attributable to the fact that a lot of awareness programs on the risks associated with smoking outnumber those about the advantages of exercise and a healthy diet. Additionally, males were less aware than females of having a close family with cancer, hepatitis B virus infection, and passive smoking as cancer risk factors. Such gaps in information might have a harmful impact on cancer diagnosis.

Individuals with a good economy were 1.51 times more aware that smoking is a cancer risk factor than persons with a bad economy. This is similar to the findings of Gizaw et al They discovered that study participants with a high level of economic position were 3.13 times more likely to be aware of cancer warning signs than those with a low level of economic status.^[14]^ Moreover, a study conducted in Denmark found that persons with a high degree of economic status were a predictor variable that impacted the level of awareness of cancer risk factors/symptoms. This means that persons with a high degree of income may have access to information regarding cancer risk factors through mass media such as televisions and the Internet and may have a better experience visiting healthcare facilities.

#### 4.1.2. Recognized symptoms

The most often recognized cancer symptom in our study was unexplained weight loss, followed by unexplained swelling/lump, and lastly unexplained pain. On the other hand, the least known cancer symptom was continuous difficulty swallowing. In contrast to our findings, earlier studies found that participants more commonly reported unexplained masses or lumps as cancer symptoms.^[7,15–17]^ Another study found that the appearance of mole followed by an unexplained lump or swelling was the most well-known indication of cancer.^[18]^ This might be related to the individuals’ diverse experiences, or to the initial symptoms that their sick relatives or friends have experienced.

In this study, men were shown to be less aware of various cancer symptoms such as unexplained swelling/lump, change in the appearance of a mole, and unexplained bleeding. Women often utilize healthcare services more frequently than males due to family planning, hormonal treatment, pregnancy, childcare, and cancer screening. Increased access to healthcare services raises health-related information and motivates women to engage in more preventative and protective activities than males, including cancer detection.^[16]^ Furthermore, the male gender role may interfere with healthy behavior, such as the idea of being less at risk of cancer, which may postpone getting medical care at an appropriate time.^[16,19]^

Nevertheless, those with a high economy were more informed of all cancer symptoms than people with a low economy, with the exception of a change in the appearance of a mole, which people with a low economy recognized more. This is similar to the findings of Hvidberg et al, who discovered a large socioeconomic gradient related to cancer awareness.^[18]^ The mechanisms behind the relation between socioeconomic status (SEP) and cancer awareness are not completely clear. It has been proposed that the association is, to some extent, associated with health illiteracy and, as a result, a decreased capacity among those with lower SEP to access, process, and interpret health information.^[20]^ As a result, our findings show that more targeted messaging is for people with lower SEP.

### 4.2. Less seeking healthcare

Fear was the most frequently stated explanation for some individuals not going to the doctor even when significant symptoms appeared, while people not wanting to waste the doctor time was the least commonly reported reason. This is similar to prior research, which found that barriers can be emotional, such as being concerned about probable outcomes, being afraid to see a doctor, and being concerned about wasting a doctor time.^[21]^ However, according to a UK benchmarking research, the most commonly supported obstacles by adults were service barriers, difficulties scheduling an appointment, and concern about wasting the doctor time.^[22]^ The causes vary by nation and culture. Understanding the reasons behind patients’ delays in seeking care is vital for informing methods for encouraging quick presentation.

Females, on the other hand, were more likely than males to identify fear as a cause. The Gaza research revealed similar results.^[8]^ Emotional constraints were reported more frequently than service or practical barriers, as predicted, and larger percentages were achieved, particularly among females. One probable explanation is that women, naturally, have a higher rate of cancer fear, denial, and dependence on alternative therapies.

Also, persons living in rural regions reported more difficulties finding a means to get to the doctor than urban residents. They also found difficulties arranging a meeting with the doctor, while those with good economic status “they do not want to waste the doctor time” by scheduling unneeded appointments. This contradicts the findings of McCutchan et al, who discovered that people with lower SEP report more emotional barriers, such as being worried or embarrassed, while people with higher SEP report more practical barriers, such as being too busy.^[23]^

## 5. Limitations

Our research has several limitations. First, because these data were from a cross-sectional design, we cannot make causal claims regarding the relationships between cancer beliefs and sociodemographic or behavioral correlates. Second, more educated participants were included in this study, which may explain the strong influence of education level on cancer risk factors and symptoms awareness. Third, since most of the assessed individuals live in urban regions, our study may not be fully representative of the Jordanian population. Finally, the survey did not record the response rate, preventing a comparison of respondents and nonrespondents.

## 6. Future directions

It is necessary to collect information depending on ethnicity and different age groups. More educational effort is required in Jordan to raise public health awareness of the need of living a healthy lifestyle in order to lower the occurrence of cancer. The public capacity to interpret spoken media messages varies, which may restrict the effectiveness of both public health campaigns and provider-patient communication. Further studies are needed to identify message features that promote good comprehension of cancer-related spoken media communications.^[24]^ Longitudinal and experimental studies would be beneficial in determining the direction of this association and whether or if increasing understanding of real causes of cancer improves health behavior choices.

## 7. Conclusion

According to the findings of this research, individuals with higher education levels demonstrated greater awareness regarding cancer risk factors, such as smoking, as well as the identification of unexplained weight loss as a potential cancer symptom. Nevertheless, public knowledge regarding cancer symptoms and risk factors remains generally inadequate, underscoring the necessity of ongoing public education, particularly regarding the correlation between lifestyle choices and cancer risk. The most commonly cited barriers for not seeking early medical attention included concerns about wasting the doctor time, emotional obstacles, and difficulties scheduling appointments with a doctor, which must be effectively addressed.

## Author contributions

**Conceptualization:** Samir Al Bashir, Majd M. AlBarakat, Ali Al-Khalaileh, Amer Alassaf, Othman Saleh, Asmaa W. Ayyad, Karem H. Alzoubi.

**Data curation:** Samir Al Bashir, Majd M. AlBarakat, Karam K. Alabedalhalim, Ali Al-Khalaileh, Amer Alassaf, Othman Saleh, Asmaa W. Ayyad, Karem H. Alzoubi

**Formal analysis:** Samir Al Bashir, Majed M. Albarakat.

**Funding acquisition:** Samir Al Bashir.

**Investigation:** Samir Al Bashir, Majd M. AlBarakat, Ali Al-Khalaileh, Amer Alassaf, Othman Saleh, Asmaa W. Ayyad, Karem H. Alzoubi.

**Methodology:** Samir Al Bashir, Majd M. AlBarakat, Karam K. Alabedalhalim, Ali Al-Khalaileh, Amer Alassaf, Othman Saleh, Asmaa W. Ayyad.

**Project administration:** Samir Al Bashir, Karem H. Alzoubi.

**Resources:** Samir Al Bashir, Karem H. Alzoubi.

**Supervision:** Samir Al Bashir, Karem H. Alzoubi.

**Validation:** Samir Al Bashir.

**Visualization:** Samir Al Bashir.

**Writing – original draft:** Samir Al Bashir, Majd M. AlBarakat, Karam K. Alabedalhalim, Ali Al-Khalaileh, Amer Alassaf, Othman Saleh, Asmaa W. Ayyad.

**Writing – review & editing:** Samir Al Bashir, Karem H. Alzoubi.
